# Efficacy and Safety of Oral Antidiabetic Drugs in Comparison to Insulin in Treating Gestational Diabetes Mellitus: A Meta-Analysis

**DOI:** 10.1371/journal.pone.0109985

**Published:** 2014-10-10

**Authors:** Nalinee Poolsup, Naeti Suksomboon, Muhammad Amin

**Affiliations:** 1 Department of Pharmacy, Faculty of Pharmacy, Silpakorn University, Nakhon-Pathom, Thailand; 2 Department of Pharmacy, Faculty of Pharmacy, Mahidol University, Bangkok, Thailand; University of Catanzaro Magna Graecia, Italy

## Abstract

**Objective:**

To assess the efficacy and safety of oral antidiabetic drugs (OADs) in gestational diabetes mellitus (GDM) in comparison to insulin.

**Methods:**

A meta-analysis of randomized controlled trials was conducted. The efficacy and safety of OADs in comparison to insulin in GDM patients were explored. Studies were identified by conducting a literature search using the electronic databases of Medline, CENTRAL, CINAHL, LILACS, Scopus and Web of Science in addition to conducting hand search of relevant journals from inception until October 2013.

**Results:**

Thirteen studies involving 2,151 patients met the inclusion criteria. These studies were randomized controlled trials of metformin and glyburide in comparison to insulin therapy. Our results indicated a significant increase in the risk for preterm births (RR, 1.51; 95% CI, 1.04–2.19, p = 0.03) with metformin compared to insulin. However, a significant decrease in the risk for gestational hypertension (RR, 0.54; 95% CI, 0.31–0.91, p = 0.02) was found. Postprandial glucose levels also decreased significantly in patients receiving metformin (MD, −2.47 mg/dL; 95% CI, −4.00, −0.94, p = 0.002). There was no significant difference between the two groups for the remaining outcomes. There were significant increases in the risks of macrosomia (RR, 2.34; 95% CI, 1.18–4.63, p = 0.03) and neonatal hypoglycemia (RR, 2.06; 95% CI, 1.27–3.34, p = 0.005) in the glyburide group compared to insulin whereas results for the other analyzed outcomes remained non-significant.

**Conclusion:**

The available evidence suggests favorable effects of metformin in treating GDM patients. Metformin seems to be an efficacious alternative to insulin and a better choice than glyburide especially those with mild form of disease.

## Introduction

Gestational Diabetes Mellitus (GDM) is defined as the diabetes occurring during pregnancy that is not clearly overt diabetes [1]. Normally, GDM ends at the termination of pregnancy; however, such patients are at a higher risk for development of type 2 diabetes mellitus later in their life [2]. GDM complicates pregnancy in many ways. It increases the risk of macrosomia, large for gestational age (LGA) births, shoulder dystocia, birth trauma, neonatal hypoglycemia, preterm births, hyperbilirubinemia and intrauterine growth retardation [3]–[7]. Later in life, such infants are at risk for disease conditions such as delayed motor development, premenopausal breast cancer, obesity and diabetes [8]–[11]. Besides type 2 diabetes, maternal adverse outcomes of GDM include increased in risk of caesarean section, induced labor, pre-eclampsia and gestational hypertension [4]. Pre-eclampsia is correlated with maternal adverse cardiovascular outcomes in the future [12].

Traditionally, GDM is treated by dietary interventions and glucose monitoring [13], [14]. Human insulin is administered to those patients for whom dietary advice fail to achieve desired glycemic goals, although prescribing of rapid acting insulin analogues (insulin lispro, insulin aspart) is also increasing [13], [14]. There has been much debate about efficacy and safety of oral antidiabetic drugs (OADs) for use in GDM patients. The National Institute for Health & Care Excellence (NICE) clinical practice guidelines recommend use of metformin and glyburide instead of insulin if life style interventions fail to control glycemic levels [13]. After a long debate, the new clinical practice guidelines of the American College of Obstetricians and Gynecologists (ACOG) also recommend use of these two agents in GDM patients as alternatives to insulin therapy and consider the combination equally efficacious [14]. But the American Diabetes Association (ADA) has not yet provided any specific recommendation for treating GDM [1]. In addition, regulatory authorities around the globe including the U.S. Food and Drug Administration (FDA) have not approved any OAD for GDM. Treatment of GDM with OADs is preferred due to low cost of therapy and ease in handling compared to insulin. In addition, adverse effects of insulin therapy, such as weight gain and hypoglycemia are problematic. However, controversies exist with regard to use of OADs during pregnancy due to limited data on efficacy and safety. Individual studies are few and do not possess adequate power to achieve statistical significance for many adverse outcomes of GDM. The reviews in the recent past have compared efficacy and safety of metformin and glyburide to insulin wherein randomized controlled trials (RCTs) of both drugs were combined and considered as a single entity [15], [16]. As a result, effects of these two different medications with different mechanisms of action could not be differentiated. Moreover, these reviews were published prior to recent studies. Keeping in mind the potential hazards of GDM and ambiguities in clinical practice of this disease, we conducted this meta-analysis to determine if OADs are as efficacious and safe as insulin in treating GDM.

## Methods

We conducted this meta-analysis according to Cochrane Handbook for Systematic Reviews of Interventions [17], and data are presented according to the recommendations of PRISMA statement [18].

### Eligibility Criteria

The eligibility criteria for the studies included in this meta-analysis were that they be randomized controlled trials (RCTs) of OADs comparing efficacy and safety parameters against insulin in GDM patients. The studies had to report at least one outcome of interest and patients had to receive an intervention after dietary advice failed to achieve desired glycemic targets. Individual study definitions of GDM were accepted and no restriction was made regarding age, parity or gravida. However, studies involving pregnant women with pre-existing diabetes were ineligible.

### Outcomes of Interest

Outcomes of interest were divided into two categories: (1) Fetal/neonatal outcomes and (2) maternal outcomes. Fetal/neonatal outcomes included macrosomia, LGA births, shoulder dystocia, birth trauma, neonatal hypoglycemia, perinatal/neonatal mortality, congenital abnormality, preterm birth and small for gestational age (SGA) babies. Maternal outcomes included caesarean section, gestational hypertension, pre-eclampsia and maternal hypoglycemia.

### Literature Search and Study Selection

For identification and selection of eligible studies, we conducted a literature search of the electronic databases, Medline (PubMed), Cochrane Central Register of Controlled Trials (CENTRAL), Cumulative Index to Nursing and Allied Health Literature (CINAHL), Latin American and Caribbean Health Sciences Literature (LILACS), http://clinicaltrialsresults.gov register, http://clinicaltrials.gov register, Scopus and Web of Science for reports of RCTs published from inception up to October 2013. Studies were identified without any language restriction. In addition, we conducted a hand search for relevant journals and conference proceedings.

We used gestational diabetes, randomized controlled trials, large for gestational age, oral antidiabetic drugs, glyburide and metformin as search terms. We also used MeSH term “gestational diabetes” for our study search.

### Data Collection

Data from individual studies were abstracted and methodological quality was evaluated independently by two authors using a standardized form, a form developed to extracts the data. Any ambiguity regarding data collection was resolved by a third author. Data was collected from individual studies for variables as age, body mass index (BMI), diagnostic criteria used to identify GDM patients, population source, threshold glycemic levels for initiating medical intervention, interventions employed, outcomes of interest and methodological quality.

### Risk of Bias

We assessed the risk of bias in individual studies using Cochrane Risk of Bias Tool [17]. Each study was assessed for selection bias, performance bias, attrition bias, reporting bias and other risk of bias according to the criteria defined in the Cochrane Handbook for Systematic Reviews of Interventions. Accordingly, domains in each study were classified as high, unclear and low risk of bias [17].

### Statistical Analysis

We used Review Manager (Rev Man 5.2.7) software for statistical analysis. The fixed effects meta-analysis model was used for combining data from individual trials in the absence of significant heterogeneity, while the random effects model was used if heterogeneity was significant. The effect measure used to present the dichotomous data was risk ratio (RR) with 95% confidence interval (CI). For continuous data we used mean difference if an outcome was measured using the same method. Inverse-variance method was used for meta-analysis because of its wide applicability and it can be used to combine both dichotomous and continuous data. Chi^2^ and I^2^ statistics were used to assess heterogeneity among studies. A low p-value i.e., <0.10 in Chi^2^ test for heterogeneity or I^2^>50% was considered an indication of substantial heterogeneity. We assessed reporting bias by using Egger’s test [19] in case five or more studies were included for an outcome.

## Results

**Figure 1:**
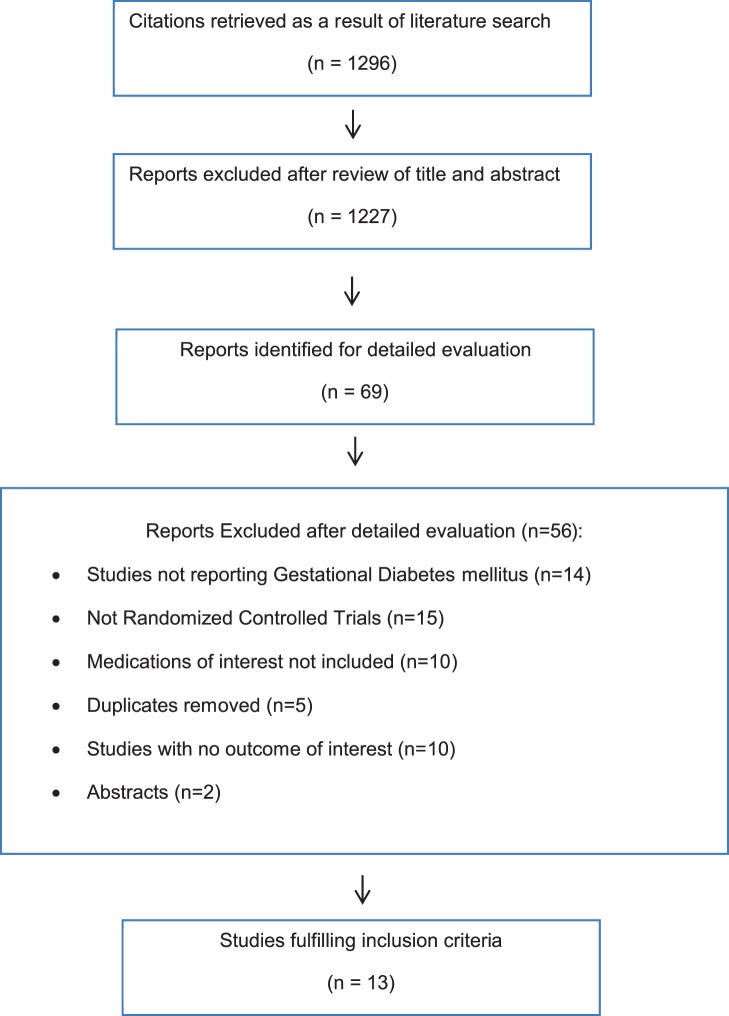
Flow chart of article selection.

Study selection process is shown in Figure 1. Our search identified 1,296 studies initially. We reviewed titles and abstracts of each study and 69 relevant studies were selected for detailed analysis. After a thorough investigation and detailed analysis, we identified thirteen relevant studies meeting our inclusion criteria which involved 2,151 patients. These studies were divided into the following two pools. Pool A included six studies that compared metformin to insulin [20]–[25]. Pool B included seven studies that compared glyburide to insulin [26]–[32]. Characteristics of studies in pool A, and B are presented in Tables 1 and 2, respectively.

**Table 1:** Characteristics of studies comparing metformin and insulin.

Study	N	Mean AgeYrs. (SD)	Mean BMI(SD)	Diagnostic Criteria	Dose of Metformin	Threshold Glycemiclevels for InitiatingMedical Intervention	Pts. on Insulin*
Moore 2007USA [21]	63	27.4 (5.7)	NA	100 g OGTT; 2 or more abnormalF≥105 mg/dL 1 h≥190 mg/dL; 2 h≥165 mg/dL; 3 h≥145 mg/dL	Starting Dose: 500 mg twice/day;Dose Titration: Dose increased as necessary;Maximum Dose: 2000 mg/day	F>105 mg/dL,2 h>120 mg/dL	0%
Rowan 2008 Australia,NZ [23]	733	33.2 (5.2)	34.8 (7.7)	75 g OGTT; Any abnormal;F>99 mg/dL; 2 h>144 m/dl	Starting Dose: 500 mg once or twice/day;Dose Titration: Dose increased over a periodof 1 to 2 weeks as necessary;Maximum Dose: 2500 mg/day	F>97.2 mg/dL;2 h>120.6 mg/dL	46.3%
Ijas 2011Finland [20]	97	32 (5.8)	31 (6)	75 g OGTT; 1 or more abnormal;F>95.4 mg/dL; 1 h>198 mg/dL;2 h>172.8 mg/dL	Starting Dose: 750 mg/day for the 1^st^ week;Dose Titration: 750 mg twice/day for 2^nd^ weekand thrice/day for 3^rd^ week;Maximum Dose: 2250 mg/day	F≥95.4 mg/dL;1.5 h≥120.6 mg/dL	31.9%
Niromanesh 2012Iran [22]	160	31.2 (5.3)	27.6 (3.0)	100 g OGTT; 2 or more abnormal;F≥95 mg/dL; 1 h≥180 mg/dL;2 h≥155 mg/dL; 3 h≥140 mg/dL	Starting Dose: 500 mg twice/day;Dose Titration: Dose increased by 500–1000 mgevery 1 or 2 weeks;Maximum Dose: 2500 mg/day	F>95 mg/dL;2 h>120 mg/dL	14%
Spaulonci 2013Brazil [24]	92	32.3 (5.3)	31.6 (5.2)	100 g OGTT or 75 g OGTT2 or more abnormal;F≥95 mg/dL; 1 h≥180 mg/dL;2 h≥155 mg/dL; 3 h≥140 mg/dL	Starting Dose: 1700 mg/day;Dose Titration: 850 mg/day for next week as necessary;Maximum Dose: 22050 mg/day	F≥95 mg/dL;2 h≥120 mg/dL	26%
Tertti 2013Finland [25]	217	32 (5.2)	29.1 (5.3)	75 g OGTT**; 2 or more abnormal;F≥86.4 mg/dL; 1 h≥180 mg/dL;2 h≥156.6 mg/dL; Or F≥95.4 mg/dL;1 h≥180 mg/dL; 2≥154.8 mg/dL	Starting Dose: 500 mg/day for initial 2 daysand twice/day for 1^st^ week;Dose Titration: Dose increased as necessary;Maximum Dose: 2000 mg/day	F≥99 mg/dL;1 h≥140.4 mg/dL	20.9

Yrs.: Years.

*Percentage of patients requiring insulin therapy in metformin group. BMI: Body mass index. OGTT: Oral glucose tolerance test. F: Fasting.

**Two different cutoff values were taken due to change in diagnostic criteria by Finnish national guidelines during course of study.

**Table 2:** Characteristics of studies comparing glyburide and insulin.

Study	N	Mean Age Yrs. (SD)	Mean BMI(SD)	Diagnostic Criteria	Dose of Glyburide	Threshold Glycemiclevels for InitiatingMedical Intervention	Pts. on Insulin*
Anjalakshi 2007India [26]	23	26.1 (4.7)	24.0 (4.3)	75 g OGTT; 2 h>140 mg/dL	Starting Dose: 0.625 mg/day;Dose Titration: Once a week to maintain2 hour, PPG<120 mg/dL	2 h≥120 mg/dL	0%
Bertini 2005Brazil [27]	51	29.9 (5.2)	27.2 (6.5)	75 g OGTT; Any abnormal;F≥110 mg/dL; 2 h≥140 mg/dL	Starting Dose: 5 mg/day;Dose Titration: Dose increased per weekas necessary; Maximum Dose: 20 mg/day	F>90 mg/dL;2 h>120 mg/dL	NA
Lain 2009USA [28]	82	31.7 (5.4)	32.1 (9.3)	100 g OGTT; 2 or more abnormal;F≥95 mg/dL; 1 h≥180 mg/dL;2 h≥155 mg/dL; 3 h≥140 mg/dL	Starting Dose: 2.5 mg/day;Dose Titration: 2.5–5 mg increase per weekas necessary; Maximum Dose: 20 mg/day	F≥95 mg/dL;2 h≥120 mg/dL	7%
Langer 2000USA [29]	404	29.5 (6.5)	≥27.3** (67%)	100 g OGTT#; 2 or more abnormal;F≥95 mg/dL; 1 h≥180 mg/dL;2 h≥155 mg/dL; 3 h≥140 mg/dL	Starting Dose: 2.5 mg/day;Dose Titration: 2.5 mg increase duringinitial week, thereafter5 mg/week as necessary;Maximum Dose: 20 mg/day	F≥95 mg/dL;2 h≥120 mg/dL	4%
Ogunyemi 2007USA [30]	97	NA	31.4 (7.2)	NA	Not reported; (Mean Final Dose: 5 mg/day)	NA	6%
Silva 2007Brazil [31]	68	30.7 (5.1)	27.7 (5.9)	75 g OGTT; Any abnormal;F≥110 mg/dL; 2 h≥140 mg/dL	Starting Dose: 2.5 mg/day;Dose Titration: 2.5 mg increase per weekas necessary; Maximum Dose: 20 mg/da	F>90 mg/dL;2 h>100 mg/dL	18.75%
Tempe 2013India [32]	64	≤30Ψ (92%)>30 (7%)	NA	100 g OGTT; 2 or more abnormal;F≥95 mg/dL; 1 h≥180 mg/dL;2 h≥155 mg/dL; 3 h≥140 mg/dL	Starting Dose: 2.5 mg/day;Dose Titration: 2.5 mg increase very three daysas necessary; Maximum Dose: 20 mg/day	F>95 mg/dL;2 h>120 mg/dL	6%

Yrs.: Years.

*Percentage of patients requiring insulin therapy in glyburide group. NA: Not available. BMI: Body mass index. F: Fasting.

**Mean BMI≥27.3 was observed in 67% of subjects included in the study.

# Only women with FPG≥95<140 mg/dL were considered eligible.

Ψ 92% of the patients enrolled in the study were having BMI≤30 and 7% had BMI>30.

### Risk of Bias Assessment

**Figure 2:**
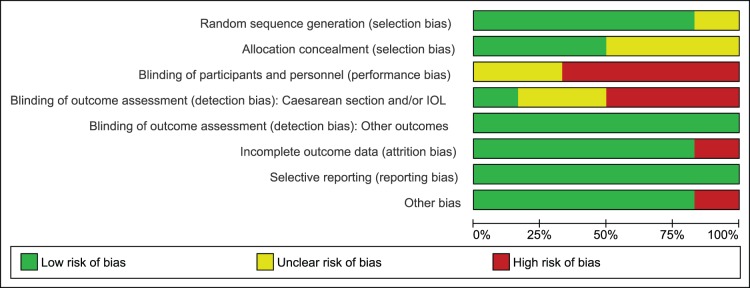
Risk of bias graph of studies comparing metformin to insulin.

**Figure 3:**
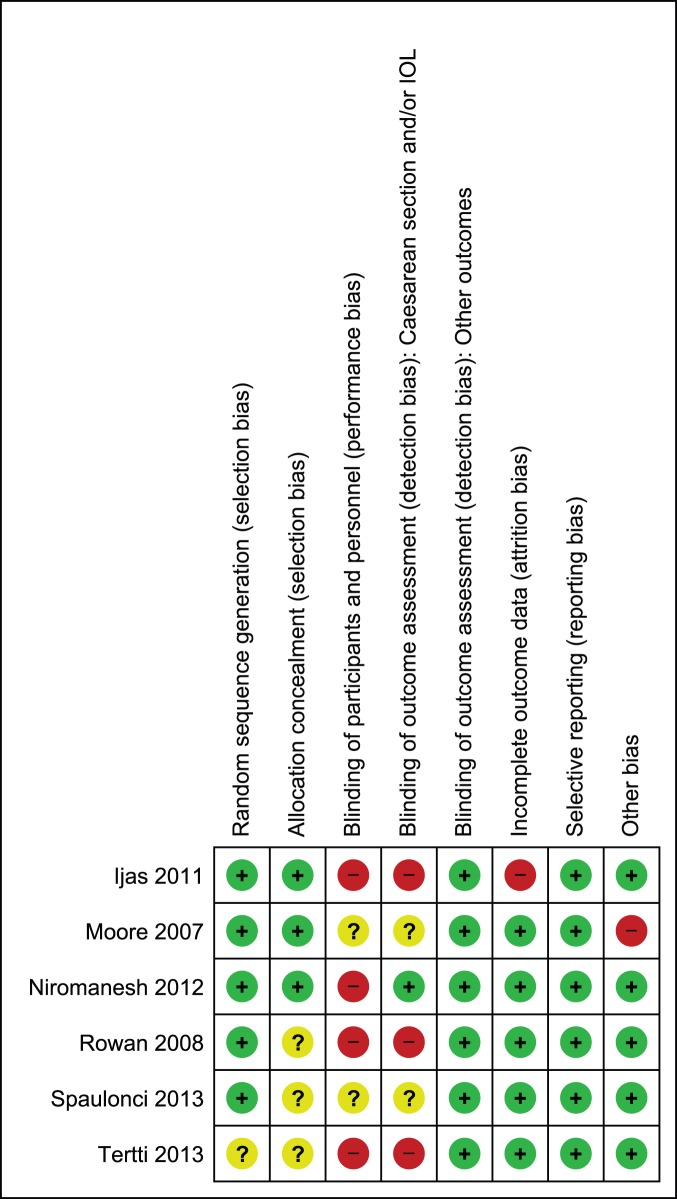
Risk of bias summary of studies comparing metformin to insulin.

In pool A, sequence generation was performed adequately in five studies [20]–[24], while in one study it remained unclear [25]. Allocation was properly concealed in three studies [20]–[22] only and in the remaining three [23]–[25] it remained unclear due to limited information provided by the authors on this domain. In four studies, blinding was not performed in a double blind fashion [20], [22], [23], [25] while in remaining two, information was not provided completely to assess the risk; therefore the domain of performance bias remained either at high or unclear risk of bias. However, double blinding was not practical due to the fact that different forms of interventions were used in the study. For blinding of outcome assessment, we found high risk or unclear risk of bias in assessing caesarean section and labor induction in most studies [20], [21], [23]–[25]. We considered lack of blinding a serious threat for validity of data for these outcomes as increase in rate of suspected macrosomia was reported to be linked with increase in risk of these two outcomes also [33]. We assigned low risk of detection bias for the rest of outcomes as lack of blinding was not likely to affect validity of data for outcomes in this category. We observed attrition in one study only [20] while selective reporting was not noticed in any study. In one study, there was baseline imbalance in the weight of patients, this study was assigned high risk for other risk of bias [21]. For the rest of studies, we observed no other bias; these studies remained at low risk. Figures 2 and 3 depict risk of bias and summary. Most of the data in this pool of our meta-analysis was from studies at low risk of bias.

**Figure 4:**
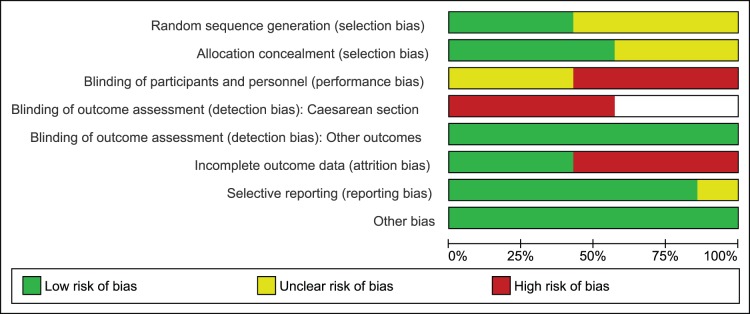
Risk of bias graph of studies comparing glyburide to insulin.

**Figure 5:**
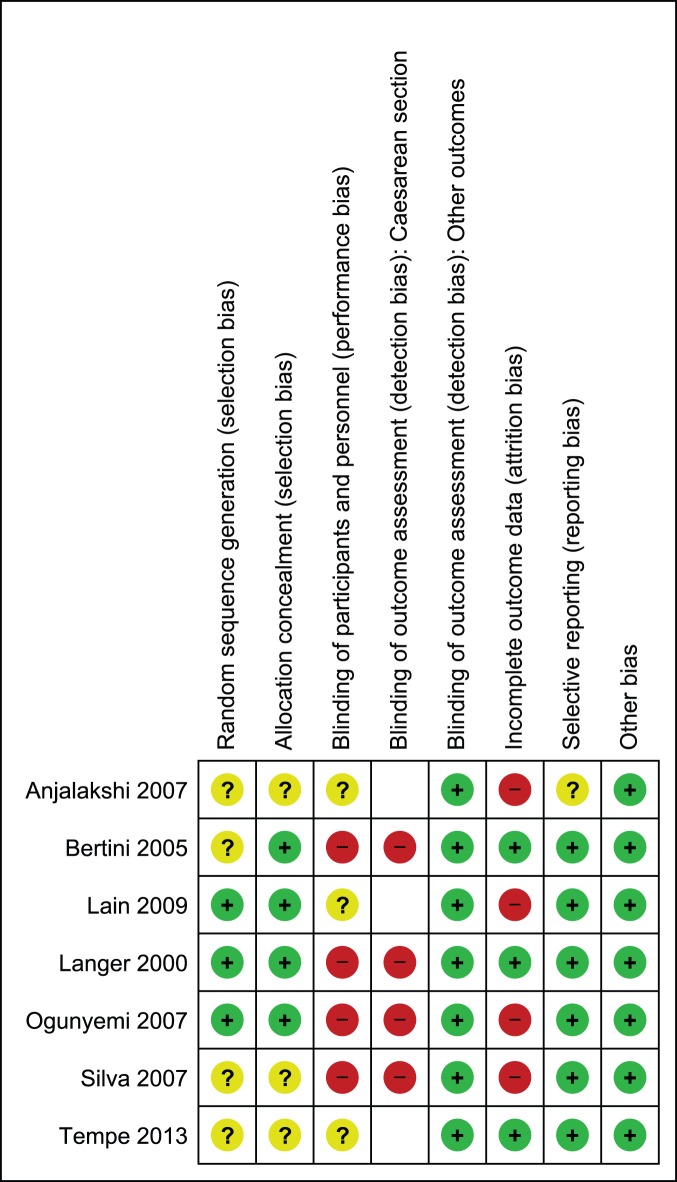
Risk of bias summary of studies comparing glyburide to insulin.

Three of the studies in pool B employed adequate sequence generation methods [28]–[30], while the remaining four studies did not provide complete information on this domain [26], [27], [31], [32]. Allocation was properly concealed in four studies [27]–[30] and remained unclear in three studies [26], [31], [32]. Similar to our earlier assessment in pool A, studies were either not blinded or information remained unclear on this domain. Therefore, risk of performance bias remained either high or unclear in included studies. For blinding of outcome assessment, we found high risk of bias in assessing caesarean section in all studies reporting this outcome as lack of blinding was likely to affect validity of data for the reasons mentioned earlier [27], [29]–[31]. While for rest of the outcomes, we did not consider lack of blinding a serious threat for outcome assessment. We observed attrition bias in four studies [26], [28], [30], [31], while three studies had complete outcome data [27], [29], [32]. Information on selective reporting remained unclear in one study only [26] while in rest, no selective reporting was noticed. No other risk of bias was observed in the studies included in pool B. Figures 4 and 5 illustrate risk of bias graph and summary. Most of the data in this pool of our meta-analysis was also from studies at low risk of bias.

### Neonatal and Maternal Outcomes

The main outcomes of interest in the majority of studies were macrosomia and LGA births. A significant proportion of studies reported on neonatal hypoglycemia and preterm births. Most often macrosomia was defined as birth weight above 4,000 g and LGA as birth weight above the 90^th^ percentile. Neonatal hypoglycemia was mostly defined as glucose levels below 40 mg/dL and prematurity was defined as birth before the 37^th^ week of gestation.

#### Pool A: Metformin versus Insulin

**Figure 6:**
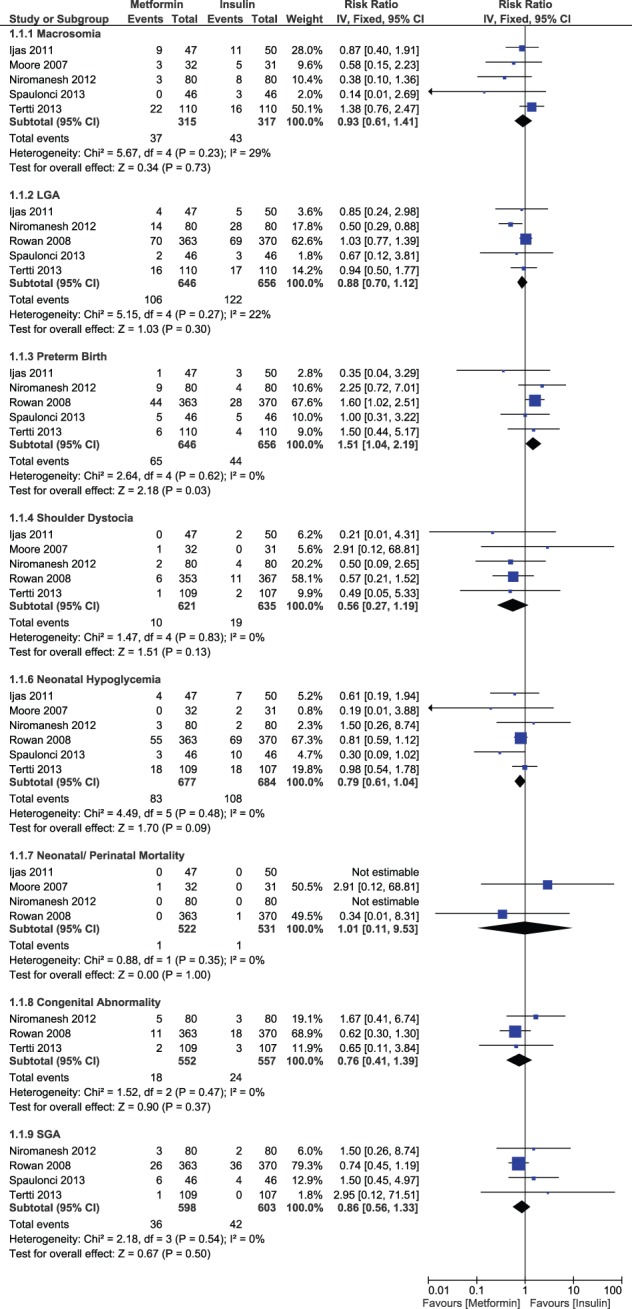
Neonatal outcomes comparing metformin and insulin.

There was a non-significant difference in the risk of macrosomia (RR, 0.93; 95% CI, 0.61–1.41) and LGA births (RR, 0.88; 95% CI, 0.70–1.12) between the two study groups. However, a significant increase in the risk of preterm births occurred in the metformin group as compared to insulin (RR, 1.51; 95% CI, 1.04–2.19, p-value = 0.03). Rate of neonatal/perinatal mortality was very low in both groups and results remained statistically non-significant (RR, 1.01; 95% CI, 0.11–9.53). Risk of shoulder dystocia (RR, 0.56; 95% CI, 0.27–1.19), neonatal hypoglycemia (RR, 0.79; 95% CI, 0.61–1.04), congenital abnormality (RR, 0.76; 95% CI, 0.41–1.39) and SGA births (RR, 0.86; 95% CI, 0.56–1.33) tended to be lower with metformin but statistical significance was not achieved (Figure 6).

**Figure 7:**
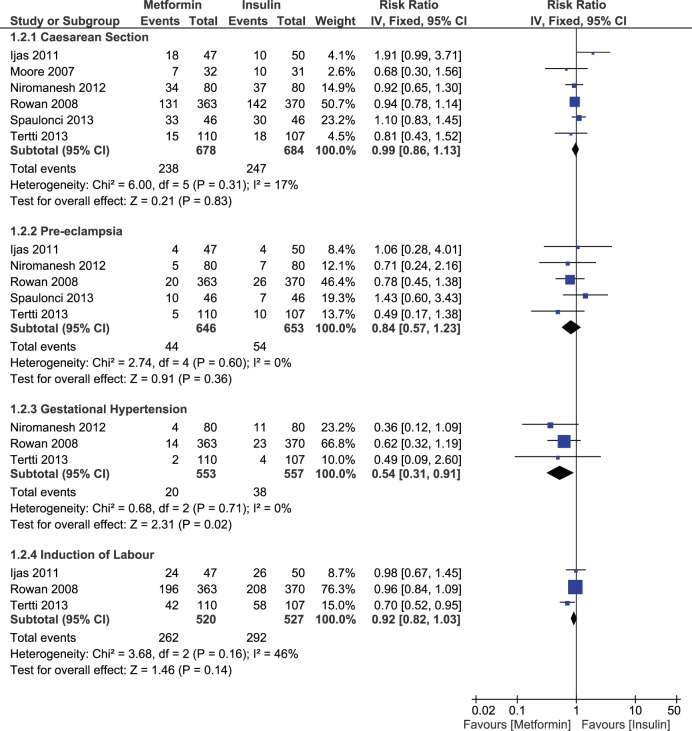
Maternal outcomes comparing metformin and insulin.

A non-significant decrease in risk of caesarean section (RR, 0.99; 95% CI, 0.86–1.13) pre-eclampsia (RR, 0.84; 95% CI, 0.57–1.23) and labor induction (RR, 0.92; 95% CI, 0.82–1.03) was noticed with metformin compared to insulin. However, we observed a significant decrease in the risk of gestational hypertension in the metformin arm (RR, 0.54; 95% CI, 0.31–0.91, p-value = 0.02). We also tried to identify any difference in maternal glycemic levels between the two groups. We noticed a significant decrease in post prandial glucose levels (MD, −2.47 mg/dL; 95% CI, −4.00, −0.94, p-value = 0.002) in metformin group compared to insulin, while results were statistically non-significant between the two groups for fasting glucose levels (MD, 0.74 mg/dL; 95% CI, −0.52, −2.01) (Figure 7). Studies remained consistent for all the outcomes with no significant heterogeneity.

#### Pool B: Glyburide versus Insulin

**Figure 8:**
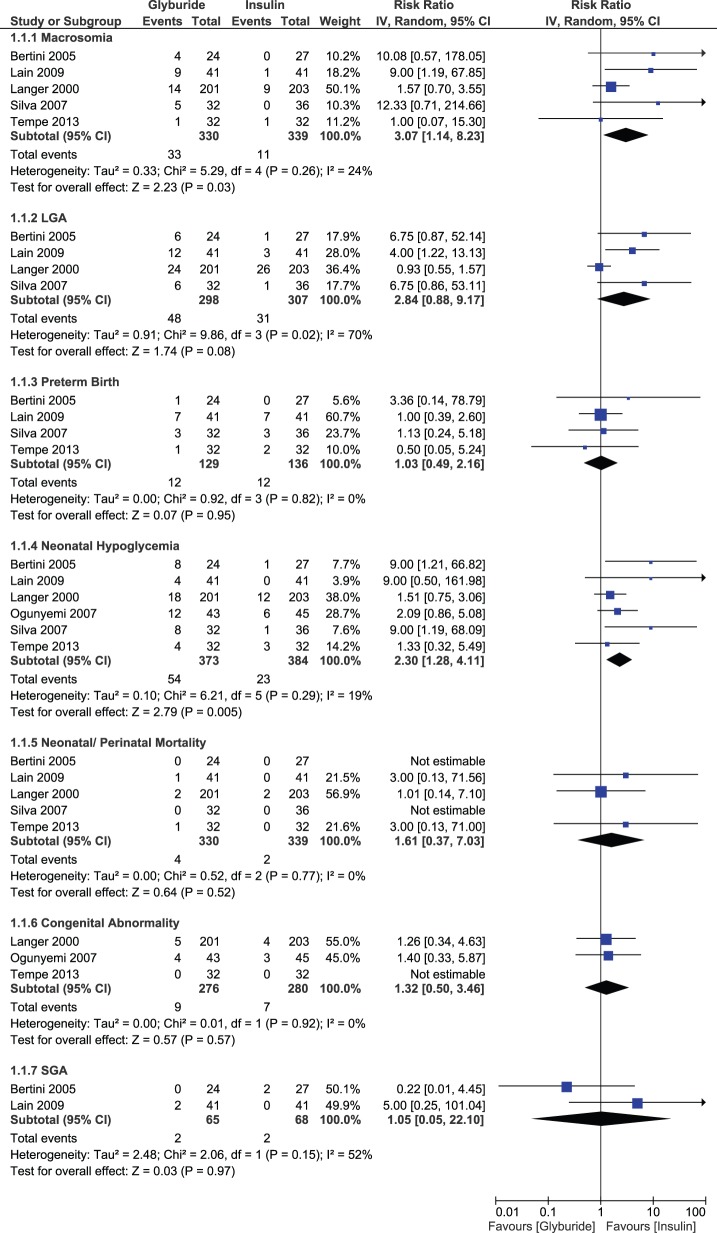
Neonatal outcomes comparing glyburide and insulin.

Glyburide significantly increased the risk of macrosomia (RR, 3.07; 95% CI, 1.14–8.23, p-value = 0.03) and neonatal hypoglycemia (RR, 2.30; 95% CI, 1.28–4.11, p-value = 0.005) compared to insulin. There was no difference between glyburide and insulin with regard to risk for LGA births (RR, 2.84; 95% CI, 0.88–9.17); statistically significant heterogeneity was detected for this outcome. There were no significant differences in the risk of preterm births (RR, 1.03; 95% CI, 0.49–2.16), neonatal mortality (RR, 1.61; 95% CI, 0.37–7.03), congenital abnormality (RR, 1.32; 95% CI, 0.50–3.46) or SGA births (RR, 1.05; 95% CI, 0.05–22.10) for glyburide versus insulin (Figure 8).

**Figure 9:**
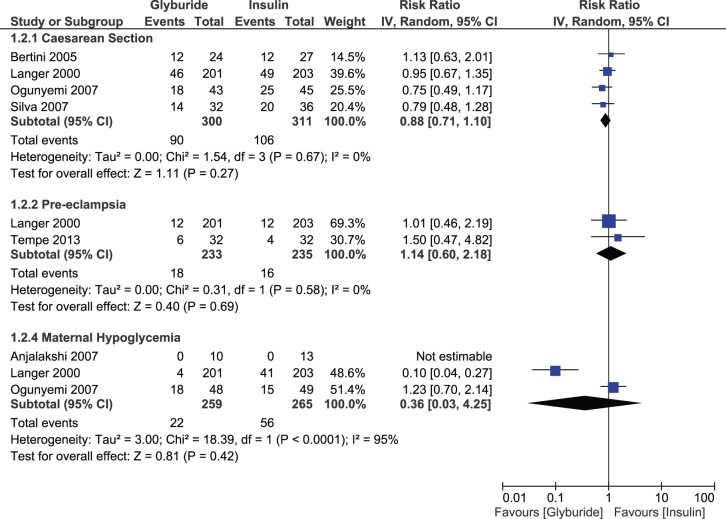
Maternal outcomes comparing glyburide and insulin.

None of the maternal outcomes displayed a significant difference between glyburide and insulin. These outcomes were caesarean section, pre-eclampsia, maternal hypoglycemia and glycemic levels. Risk of caesarean section (RR, 0.88; 95% CI, 0.71–1.10) and maternal hypoglycemia (RR, 0.36; 95% CI, 0.03–4.25) tended to be lower with glyburide, but statistical significance was not achieved. A risk of pre-eclampsia (RR, 1.14; 95% CI, 0.60–2.18) did not differ between groups. The effect estimate for fasting glucose levels (MD, 1.90 mg/dL; 95% CI, −0.38, 4.18) and postprandial glucose levels (MD, 3.42 mg/dL; 95% CI, −1.17, 8.02) favored the insulin group, but results remained non-significant (Figure 9). There was statistically significant heterogeneity for LGA births, maternal hypoglycemia and postprandial glucose levels only. Outcomes for the remaining studies were consistent and no-significant heterogeneity was observed.

### Additional Analyses

We investigated publication bias in pools A and B using Egger’s regression. In pool A, outcomes evaluated for publication bias were macrosomia, LGA births, neonatal hypoglycemia, preterm births, caesarean section and pre-eclampsia. Among all the evaluated outcomes, publication bias was detected for macrosomia only (Egger’s test: intercept, −2.27; 95% CI, −3.86, −0.67, p-value = 0.02). After making adjustment for publication bias using the trim and fill method, no difference was observed in the point estimate for macrosomia (RR, 0.93; 95% CI, 0.61–1.41).

In pool B, we evaluated macrosomia and neonatal hypoglycemia for publication bias. Publication bias was detected for neonatal hypoglycemia (Egger’s test: intercept, 1.88; 95% CI, 0.13–3.64, p-value = 0.04). After making adjustment for publication bias using the trim and fill method, risk for neonatal hypoglycemia still remained significantly higher with glyburide compared to insulin (RR, 1.95; 95% CI, 1.04–3.67).

## Discussion

Pharmacological interventions are generally initiated in GDM management when dietary therapy along with glucose monitoring fails to control desired glycemic levels. The majority of GDM patients achieve currently defined glycemic goals with dietary modification. However, a significant proportion of patients require a pharmacological agent in addition to dietary intervention. Consensus exists about the use of insulin in such patients; however, acceptability of OADs is also on the rise. We found that the studies mostly included metformin and glyburide as OADs of interest. Concern about the use of OADs in pregnancy is mostly related to first generation sulfonylurea drugs, which were reported to increase risk for fetal anomalies and neonatal hypoglycemia [34], [35]. OAD therapy is preferred in pregnancy due to ease of administration and lower cost of therapy compared to insulin. The main apprehension about the use of metformin in pregnancy is its ability to cross the placenta [36]. Since metformin is not known to be teratogenic, it becomes an OAD of interest for use in GDM [37], [38]. Glyburide, on the other hand, seems attractive since it exhibits minimal placental transport [39]. But the few RCTs with metformin and glyburide are limited by small sample size so that a firm conclusion regarding safety and efficacy cannot be established.

Our comparison of metformin and insulin revealed no difference in risk of many of the outcomes of interest, but the risk for preterm births was significantly higher for metformin. On the other hand, there was a significant reduction in the risk of gestational hypertension and postprandial glucose levels in the metformin patients. Our findings, which are based on the inclusion of more recent studies, are in agreement with an earlier meta-analysis [40]. With respect to beneficial actions of metformin, it is important to note that a significant proportion of patients in the metformin arm were shifted to insulin during course of therapy. It is difficult to determine whether this enhanced response is solely attributable to metformin or if insulin also contributed to the improvement. A decreased risk for gestational hypertension may have resulted from decreased inflammatory response and decreased insulin resistance via complex actions of metformin on maternal endothelial cell [41]. Yet, there is no plausible explanation for the increase in the risk for preterm births. Consequently, the safety of metformin in GDM patients remains questionable. Incidence of preterm births reported with metformin use in some of the studies were mostly at 33–35 week of gestation [20], [23] which may be an acceptable risk comparing adverse outcomes of hyperglycemia during pregnancy especially in a situation e.g. developing countries where availability of insulin could be an issue. Nevertheless, results of an earlier study in polycystic ovary syndrome (PCOS) patients reported a decreased incidence of preterm deliveries in comparison to placebo [42]. A greater proportion of patients in the metformin arm also experienced gastrointestinal adverse events which was not unexpected. Moreover, the long term effects of metformin on infants born to GDM mothers are unknown. Nevertheless, it is important to note that in an 18 month study of 126 infants born to PCOS mothers with who were treated with metformin, the drug had no adverse effect on their motor or social development [43].

Our meta-analysis of glyburide and insulin revealed a significant increase in the risk of macrosomia and neonatal hypoglycemia in the glyburide group. Statistically significant heterogeneity prevented analysis of the risk for LGA birth. The outcomes for other differences between the two groups remained non-significant. Of potential importance is maternal hypoglycemia, which displayed a non-significant decrease with glyburide, but very few studies addressed this important outcome of interest for either metformin or glyburide trials. Maternal hyperglycemia is mainly linked with increased risk of large babies and neonatal hypoglycemia, but in our study the difference between the two groups remained non-significant for maternal glycemic levels [44], [45]. Another factor that contributes to increased risk of macrosomia is maternal adiposity, which in our study was comparable in both groups [46]. Although, Langer et al. [29] did not detect glyburide in cord serum of infants born to GDM mothers receiving glyburide, apprehensions still exist about the transfer of drug across the placenta even in minimal quantities [39], and also in a recent pharmacokinetic study in GDM patients wherein umbilical cord concentration of drug averaged 70% of maternal concentration [47]. Higher incidence of neonatal hypoglycemia and macrosomia may be the result of even minimal transfer of drug, which might have increased insulin release from fetal beta cells. If such is the case, then this could have serious consequences for the infant in the long term due to beta cell apoptosis induced by sulfonylurea therapy. However, further studies with large sample sizes are required to confirm these findings. There is one report that infants born of overweight women and obese GDM women, who achieved glycemic control with insulin exhibited comparable rates of macrosomia as infants of normal weight GDM women. This may be due to the effects of insulin on lipid metabolism as opposed to control of glucose [46].

### Strengths and Limitations

Our review is the largest and the most updated review conducted on the topic. It includes both metformin and glyburide studies which were analyzed separately in comparison to insulin therapy. Our findings, which are based on the inclusion of more recent studies, are in agreement with the previous reviews of Dhulkotia et. al. and Gui et. al. [15], [40], however, our results are more reliable due to its increased sample size. Dhulkotia et. al. [15], combined effects of both glyburide and metformin treatment in a single group and compared jointly to insulin which made it impossible to differentiate between the actions of these two OADs acting in a different way. Further, studies published after 2009 are not part of this review. As we addressed this issue in our meta-analysis therefore, reader of our review is in a better position to develop an understanding of OAD therapy in GDM patients. We found that the use of glyburide in GDM increases the risk of macrosomia and neonatal hypoglycemia in comparison to insulin. These data are similar to a report by Zeng et al. [48] Studies that formed the basis of the analysis were consistent for the majority of outcomes and heterogeneity remained non-significant except for very few outcomes. After making adjustment for publication bias, no substantial change in the effect estimates was observed. However, we could not provide sufficient evidence in the cases of shoulder dystocia and birth injuries since the majority of studies failed to report these outcomes. Further, most of the studies were of small sample size so that statistical significance was not achieved for many low incidence outcomes. Studies in both pools did not provide evidence on the long term effects of OAD therapy both for the mother and infant. Another limitation was the lack of blinding which increased the risk of both performance and detection bias. This was especially the case for caesarean section and labor induction. In addition, there was a wide variation in diagnostic criteria, which increased the chances of variability among patients.

## Conclusion

This meta-analysis provides findings about efficacy and safety of metformin and glyburide in comparison to insulin. Our results indicate that treatment of GDM with these two OADs is likely to increase risk for some important outcomes of interest. These outcomes have important negative consequences for the infant and mother both in the short and long term. Decrease in the risk of gestational hypertension and postprandial glucose levels in patients receiving metformin is likely to have beneficial effects for both mother and infant. But the risk versus benefit must be clarified because of the increased in risk of preterm births. Glyburide increased risk of macrosomia and neonatal hypoglycemia which seems to be an adverse event independent of glycemic levels in maternal circulation. Between the two OADs studied in our meta-analysis, metformin seems to be superior to glyburide and an efficacious alternative to insulin therapy especially in patients with mild form of disease. Further studies with adequate sample size are required to confirm our findings and to determine the impact of OAD therapy on many low incidence pregnancy outcomes.

## Supporting Information

Checklist S1 **PRISMA checklist.**
(DOC)
